# Calibration for a count rate-dependent time correlation function and a random noise reduction in pulsed dynamic light scattering

**DOI:** 10.1007/s44211-022-00071-0

**Published:** 2022-02-15

**Authors:** Takashi Hiroi, Sadaki Samitsu, Hideaki Kano, Kunie Ishioka

**Affiliations:** 1 National Institute for Materials Science, 1-2-1 Sengen, Tsukuba, Ibaraki 305-0047 Japan; 2 Department of Chemistry, Faculty of Science, Kyushu University, 744 Motooka, Nishi-ku, Fukuoka, 819-0395 Japan

**Keywords:** Dynamics light scattering, Pulsed laser, Clipping effect, Noise reduction, Time gating, Photon correlation

## Abstract

A pulsed dynamic light scattering (DLS) system, which would be potentially applied to nonlinear DLS with molecular selectivity, was developed by combining a sub-nanosecond pulsed laser with a software-based detection system. The distortion of the time correlation function due to the clipping effect in the photon counting module, and the resulting underestimation of the particle size, were successfully calibrated based on a theoretical simulation. The effective removal of random noises was also demonstrated via time gating synchronized to the laser pulses.

**Graphical abstract:**

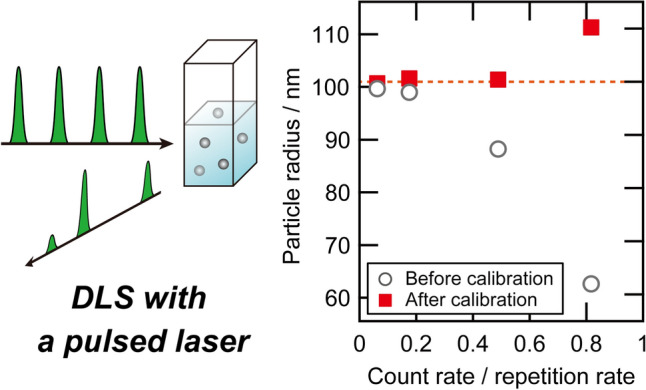

**Supplementary Information:**

The online version contains supplementary material available at 10.1007/s44211-022-00071-0.

## Introduction

Dynamic light scattering (DLS) is widely used to determine the size distributions of nanometer-scale objects in dispersions [1, 2], the mesh size of gels [3], the zeta potential of the colloidal particles [4], and the aspect ratio of rod-like particles [5] by measuring the time correlation function of the scattered light intensity. The conventional DLS uses a continuous wave (CW) laser as a light source, and the time correlation function is obtained using an autocorrelator. Recently we have developed a software-based DLS system [6], in which the arrival times of all the scattered photons are recorded [7–9]. This has enabled the application of DLS even to a dispersion containing large pollutants by calculating the time correlation function exclusively from the uncontaminated parts of the data in the post-processing. This noise reduction scheme can only be applied to transient noise; however, a new technique to effectively remove random noises, such as dark counts from the detector and the signals from background light, has not been achieved.

**Fig. 1:**
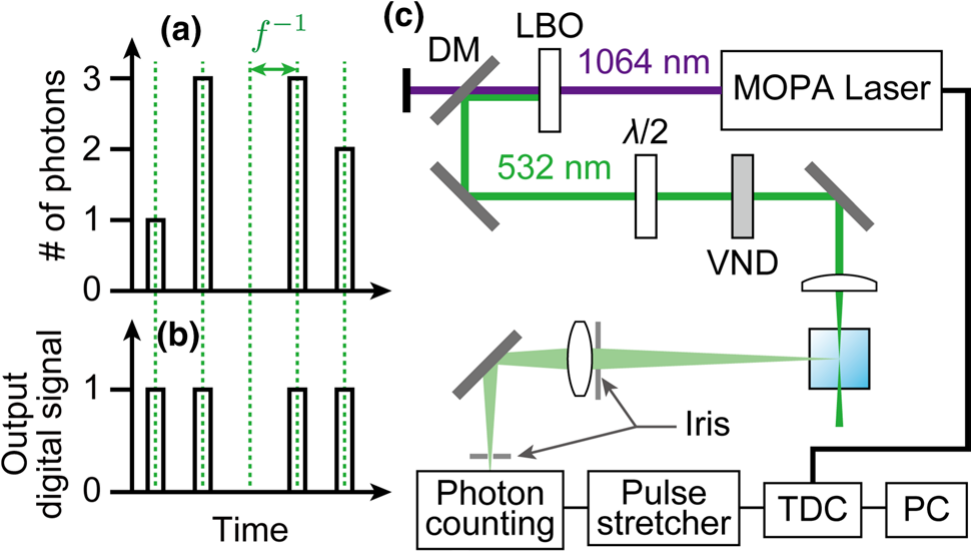
**a**, **b** Schematics comparing the actual number of photons arriving at the photon counting module (**a**) and output of the photon counting module (considering the clipping effect) **b** for pulsed DLS using a laser of repetition rate *f*. **c** Schematic of the pulsed DLS measurement apparatus. DM, dichroic mirror; VND, variable neutral density filter; TDC, time-to-digital converter

Employing a pulsed laser with a high peak intensity as the light source for DLS would realize molecular selective DLS based on nonlinear optical processes, such as hyper-Rayleigh scattering [10, 11] and coherent anti-Stokes Raman scattering [12]. In a novel attempt at pulsed DLS using a femtosecond laser as the light source [13], a *nonlinear* DLS measurement was not successful, predominantly because of the instability of the laser output. Moreover, even a *linear* pulsed DLS had a significantly worse signal-to-noise ratio than that obtained with a CW laser. This was attributed to destructive interference among the spectrally broad scattered light. In addition, the nanoparticle size obtained from the linear pulsed DLS was underestimated for some unknown reason [13]. We speculate that the underestimation originated from a false detection of the number of scattered photons. Because of the high peak intensity of the incident laser pulse, it is highly probable that more than one scattered photon arrived at a photon counting module within the pulse duration time (Fig. 1a), which was shorter than the dead time of the module. In this case, the photon-counting module would fail to count the second and subsequent photons arriving within the dead time (Fig. 1b). This is known as a clipping effect [2]. This clipping effect affects both linear and nonlinear pulsed DLS.

This paper reports on the nanoparticle size estimation using a linear pulsed DLS system, combining a sub-nanosecond laser with the software-based DLS system developed in our previous study [6]. Use of the narrowband pulsed laser enabled us to suppress destructive interference. We found that the time correlation function depends on the count rate of the scattered light on the photon counting module, leading to a systematic underestimation of the particle size in dispersion, in accordance with the previous study [13]. Our numerical simulation quantitatively reproduced the count rate dependence by considering the clipping effect, thereby giving an effective calibration to recreate the undistorted time correlation function and the precise particle size. Taking advantage of the pulsed light source, we further demonstrated an effective reduction of random noises via time-gating of the detected signals.

## Experimental

All of the DLS measurements were performed at room temperature (23 °C). Monodisperse silica nanoparticles (803847-1ML, Sigma-Aldrich, guaranteed particle concentration: 1.2 × 10^13^/mL), whose average particle radius is estimated to be 101 ± 6 nm (202 nm in diameter) evaluated by transmission electron microscopy (TEM), were used as the dispersion sample. A representative TEM image of the silica nanoparticles and the probability density of the observed nanoparticle radius are shown in Fig. S1 in Supporting Information. The dispersion was diluted using pure water to obtain 1 × 10^4^ particles/nL (1 nL is a typical irradiated volume viewed by a detector).

The developed pulsed DLS apparatus is schematically shown in Fig. 1c. The vertically polarized output of a master oscillator power amplifier (MOPA) laser (STA-01-MOPA-2, Standa, Lithuania), with a wavelength of 1064 nm, a pulse width of 400 ps, a repetition rate of *f* = 50 kHz, and a pulse energy of 50 μJ/pulse, was frequency-doubled by a lithium triborate crystal and used as a light source. The visible light pulse was focused onto a quartz cell filled with a sample dispersion after its polarization was rotated vertically by a half-wave plate. The scattered light was collected at 90° and focused onto a photon counting module (C11202-050, Hamamatsu Photonics, Japan). The dead time of the module was 15 ns. The dark count rate of the module was less than 10 counts per second (cps), which was negligibly small compared to the typical count rate of the scattered photon signal, > 10^3^ cps. The count rate at the photon counting module was varied by adjusting the incident laser intensity with the neutral density filter. For a comparison, a CW Nd:YAG laser was also used at a wavelength of 532 nm (0532-04-01-0100-700, Cobolt, Sweden). Each measurement involved the detection of 10^6^ scattered photons.

The electronic signal pulses from the photon counting module were stretched by a homebuilt pulse stretcher circuit and recorded by a time-to-digital converter (TDC, NI-9402 & cDAQ-9174, National Instruments). The arrival times of detected photons were converted into a normalized time correlation function, *g*^(2)^(*τ*), as described in our previous paper [6]. The electronic signal pulses from the MOPA laser synchronized to the laser firing time were also recorded by the TDC.

## Results and discussion

### Evaluation of nanoparticle size in dispersion by pulsed DLS system

**Fig. 2:**
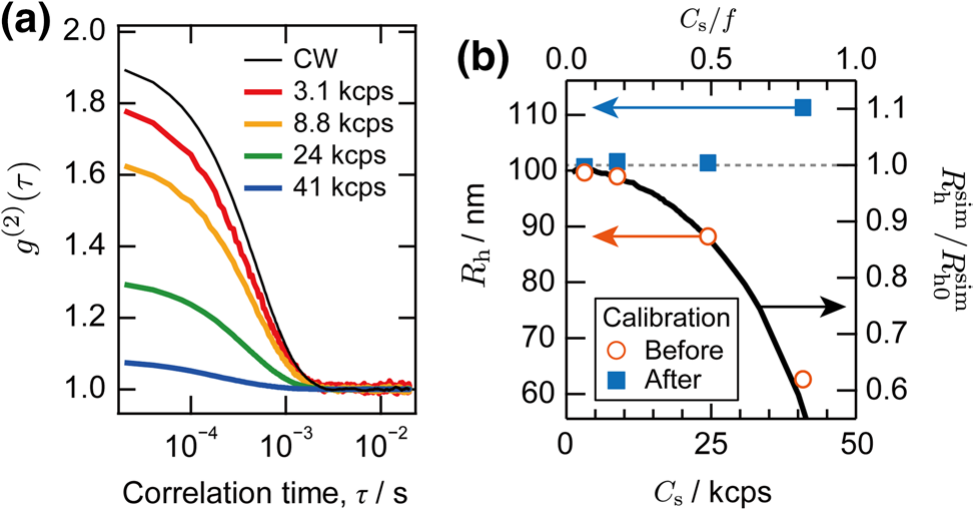
**a** Time correlation functions of the silica nanoparticle dispersion obtained from the pulsed DLS system at various count rates. The time correlation function obtained with a CW laser is also shown for a comparison. **b** Hydrodynamic radius obtained from pulsed DLS measurements before and after calibration as a function of the count rates (symbols, plotted to the left axis). The horizontal dashed line indicates the actual radius of the particle obtained from TEM (101 nm). Corresponding correction factor, *R*_h_^sim^/*R*_h0_^sim^, obtained from simulation is also plotted with a solid curve to the right axis

We first evaluated the silica nanoparticle size in the dispersion with the pulsed DLS system. Figure 2a compares the normalized time correlation function of the scattered photons from the same dispersion obtained with a pulsed laser (colored curves) and a CW laser (black curve). The time correlation obtained with the pulsed laser deviates from that obtained with the CW laser. Moreover, the deviation becomes more significant with increasing incident laser intensity, thereby increasing the count rate, *C*_s_, on the photon counting module.

In the CW DLS, the normalized time correlation function of the scattered light intensity can be expressed as

1 $$g^{(2)} (\tau ) = \beta_{0} e^{{ - 2D_{0} q^{2} \tau }} + 1,$$

where (2*D*_0_*q*^2^)^−1^ is the relaxation time and *β*_0_ is a coherence factor [2]. *D*_0_ is the diffusion constant, and *q* is the momentum transfer, which is defined as

2 $$D_{0} = \frac{{k_{{\text{B}}} T}}{{6\pi \eta R_{{{\text{h}}0}} }},$$

3 $$q = \frac{{4\pi n_{{\text{r}}} }}{{\lambda_{0} }}\sin \frac{\theta }{2},$$

where *k*_B_, *T*, *η*, *R*_h0_, *n*_r_, *λ*_0_, and *θ* are the Boltzmann constant, absolute temperature, viscosity of the solvent, hydrodynamic radius, solvent refractive index, laser wavelength in vacuum, and the scattering angle, respectively. By fitting the time correlation function obtained with the CW laser to Eq. (1), the coherence factor for the present detection setup was obtained to be *β*_0_ = 0.9. *R*_h0_ was estimated to be 104 ± 3 nm, in good agreement with the particle size obtained from TEM. We emphasize that all these quantities should be independent of the incident laser intensity and the count rate, *C*_s_.

**Fig. 3:**
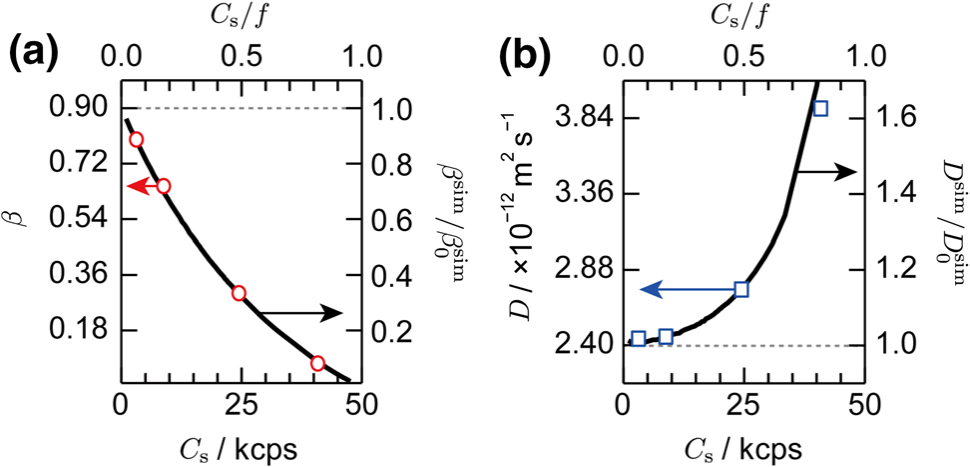
Count rate dependence of the initial amplitude (**a**) and diffusion coefficient (**b**) obtained from pulsed DLS experiments (open symbols, plotted to the left axis), and corresponding correction factors, *β*^sim^/*β*_0_^sim^ (**a**) and *D*^sim^/*D*_0_^sim^ (**b**), obtained from a numerical simulation (curves, plotted to the right axis). Horizontal dashed lines indicate the actual values

For the pulsed DLS measurements, the initial amplitude, *β*, of the correlation function, which is defined by *g*^(2)^(*τ* → 0) ≡ 1 + *β* and would correspond to the coherence factor for the CW DLS, became smaller as the count rate increased, as plotted in Fig. 3a. The nominal diffusion coefficient, *D*, obtained by fitting to Eq. (1), increased simultaneously, as shown in Fig. 3b. The latter trend resulted in the nominal particle size, *R*_h_, estimated from Eq. (2), becoming significantly smaller than the actual particle size, as shown in Fig. 2b with open circles.

A possible origin of this count rate dependence is the clipping effect. To confirm this hypothesis, we performed a numerical simulation that considered the clipping effect, whose details and flow charts (Figs. S2, S3 and S4) are shown in the Supporting Information. We considered 1 × 10^4^ particles of an identical size *R*_h0_ = 101 nm that were random walking with the diffusion constant, *D*_0_ = *k*_B_*T*/6π*ηR*_h0_. We assumed that a light electric field, $E_{{\text{i}}} (\overrightarrow {r} ,t)$, with a wavevector of $\overrightarrow {k}_{{\text{i}}}$ was incident onto the particles:

4 $$E_{{\text{i}}} (\overrightarrow {r} ,t) = E_{{\text{i}}}^{0} e^{{i(\overrightarrow {k}_{{\text{i}}} \cdot \overrightarrow {r} - \omega t)}} ,$$

where $\left| {\overrightarrow {k}_{{\text{i}}} } \right| = 2\pi n_{{\text{r}}} /\lambda_{0}$ and $\omega = c\left| {\overrightarrow {k}_{{\text{i}}} } \right|$. Here, we assumed that *E*_i_^0^ was time-independent, because it varied slowly compared with the carrier wave, $e^{{i(\overrightarrow {k}_{{\text{i}}} \cdot \overrightarrow {r} - \omega t)}}$. The electric field scattered by the particles at an angle of *θ* = 90°, *E*_s_(*t*), could then be calculated as

5 $$E_{{\text{s}}} (t) = E_{{\text{i}}}^{0} e^{ - i\omega t} \sum\limits_{j = 1}^{10000} {e^{{i\overrightarrow {q} \cdot \overrightarrow {r}_{j} (t)}} } ,$$

where $\overrightarrow {r}_{j} (t)$ is the position of the *j*th particle and $\overrightarrow {q} \equiv \overrightarrow {k}_{{\text{i}}} - \overrightarrow {k}_{{\text{s}}}$ is the momentum transfer, with $\overrightarrow {k}_{{\text{s}}}$ being the wavevector of the scattered light. The calculated scattered light intensity, $I(t) = \left| {E_{{\text{s}}} (t)} \right|^{2}$, was then converted into the number of photons, *n*(*t*, Δ*t*), detected between time *t* and *t* + Δ*t*, such that the probability followed the Poisson distribution:

6 $$P(n,t,\Delta t) = \frac{{e^{aI(t)\Delta t} (aI(t)\Delta t)^{n} }}{n!},$$

where *aI*(*t*)Δ*t* denotes the number of photons arriving at the detector between time *t* and *t* + Δ*t*. In this simulation, the number of the arriving photons was adjusted by varying the coefficient, *a*. When the signal clipping effect was neglected, the count rate of the detector was given by *C*_s_ = *aI*(*t*). In this case, the time correlation function calculated from *n*(*t*, Δ*t*) was independent of *C*_s_, as shown in Fig. S5a in the Supporting Information. The calculation agreed with the experimental time correlation function obtained from the CW DLS.

The signal clipping effect was introduced in the simulation by counting only the first photon and ignoring the rest for a given time interval, Δ*t*. This led to the number of counted photons being smaller than the arriving photons, *C*_s_ < *aI*(*t*). Figure S5b compares the calculated time correlation functions with the clipping effect for different count rates. The results reproduce the experimental results well, including the count rate dependence.

In the present simulation, the interval was set to be Δ*t* = *f* ^−1^ = 20 μs to match the experimental interval of the laser pulses. The simulation can be applied to a pulsed DLS system with any laser repetition rate *f*, as long as the dead time of the detector is shorter than the interval of the laser pulses. The disparity between the number of counted photons and that of the arriving photons depends on the ratio *C*_s_/*f* in the presence of the clipping effect. By fitting the numerically calculated time correlation function to Eq. (1), we obtained the simulated initial amplitude, *β*^sim^, and the diffusion coefficient, *D*^sim^, as a function of *C*_s_/*f* for the pulsed DLS. We also obtained the ratios *β*^sim^/*β*_0_^sim^ and *D*^sim^/*D*_0_^sim^, with *β*_0_^sim^ and *D*_0_^sim^ being the simulated initial amplitude and diffusion coefficient without considering the clipping effect, respectively, as shown with curves in Fig. 3. From Eq. (2), the corresponding ratio for the hydrodynamic radius can be expressed by *R*_h_^sim^/*R*_h0_^sim^ = *D*_0_^sim^/*D*^sim^, as shown with a curve in Fig. 2b. The calculation reproduced the experimental underestimation in the particle size quantitatively at the high-count rate, confirming that the clipping effect is the origin of the underestimation.

According to the simulation results, it is in principle desirable to measure the pulsed DLS at the lowest count rate possible to minimize the clipping effect. In the case that the high-count rate is not avoidable, however, *β*^sim^/*β*_0_^sim^ and ﻿ *D*^sim^/*D*_0_^sim^ can be used to calibrate the “clipped” experimental time correlation function, as demonstrated in Fig. S5c. The numerical values for the calibration factors are listed in Table S1 in the Supporting Information. Likewise, the underestimated experimental hydrodynamic radius can be calibrated, whose result is shown in Fig. 2b with filled squares. We emphasize that our calibration scheme does not depend on the sample condition, such as the particle size and concentration, because the origin of the distortion in the time correlation function is the clipping effect, which is purely the issue of detection. This is demonstrated by comparing the simulation results for different particle sizes, as shown in Fig. S6 and Table S2 in the Supporting Information. The calibrated hydrodynamic radius successfully reproduced the actual particle size with a precision of better than 1% for the relatively low count rate regime of *C*_s_/*f* < 0.5. In the high-count rate regime, e.g., at *C*_s_/*f* = 0.82 (41 kcps), the calibrated *R*_h_ was too large compared with the actual particle size by 10%. The remaining discrepancy may be attributed to too many (more than 80%) signals being clipped. In this case, the time correlation function deviated from Eq. (1) and was difficult to reconstruct. We, therefore, concluded that the results of the pulsed DLS measurements can be safely calibrated below the count rate of *C*_s_/*f* = 0.5.

### Random noise reduction by the pulsed DLS system

**Fig. 4:**
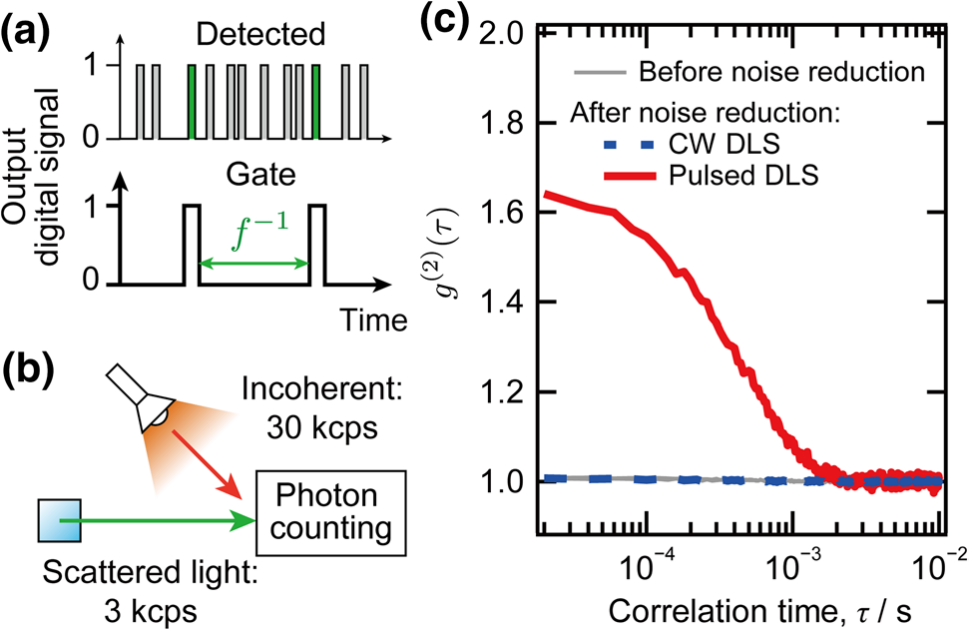
**a** Scheme for random noise reduction in the pulsed DLS using time gating, **b** Schematic of the experimental setup to demonstrate the random noise reduction, **c** Comparison of time correlation functions of time-gated (red solid line) and ungated (grey line) signals measured with a pulsed laser at count rates of scattered light and background light of 3 and 30 kcps, respectively. The time correlation function measured with a CW laser (blue dashed line) after noise reduction is also compared

In the above experiments, we did not set a time gate for the photon counting module, because it had a sufficiently low dark current. In case the DLS signal suffers from random noise, we can set a time gate to exclusively detect the scattered photons that are synchronized to the incident laser pulses (Fig. 4a). Here, we demonstrate the removal of the random noise by intentionally introducing intense incoherent light into the detector during the DLS measurement, as shown schematically in Fig. 4b. The count rates of the signal light scattered from the nanoparticle dispersion and the incoherent light source were 3 and 30 kcps, respectively.

Figure 4c compares the time correlation functions reconstructed from the ungated signal (gray line) with that from the time-gated signal (red curve), in which the gate is synchronized with the incident laser pulse with a precision of ± 25 ns. The *ungated* signal gives a time correlation function that equals unity at all correlation times, indicating that there is no correlation in the detected signals, and that it is contributed by random noise. The *time-gated* signal, in contrast, gives a time correlation function that follows a clear decay curve. Fitting the latter time correlation function to Eq. (1) yielded a hydrodynamic radius of 101 ± 1 nm after the calibration described above, which was in good agreement with the actual size obtained by TEM (101 ± 6 nm). We also performed the measurement using a CW laser at the same signal and noise count rates for a comparison. As shown with a blue dashed line in Fig. 4c, the result is similar to that of an ungated signal with a pulsed laser, even though the transient noises are removed by the post-processing noise reduction scheme proposed in our previous paper [6]. The comparison demonstrates the efficiency of the random noise removal by combining a pulsed light source and time-gated detection synchronized to it.

## Conclusions

We demonstrated a pulsed DLS system using a narrowband pulsed laser and software-based detection. This scheme allowed us to obtain an accurate particle size at a relatively low count rate, once the clipping effect of the photon counting module was calibrated. The obtained knowledge is essential for developing a novel nonlinear DLS technique using a similar pulsed light source, which would provide various selectivities to the conventional DLS. For example, DLS combined with hyper-Rayleigh scattering could exclusively monitor non-centrosymmetric molecules, whereas that with coherent anti-Stokes Raman scattering could estimate the respective particle sizes of multi-component colloidal dispersions. We also demonstrated that, even in the presence of intense random noise, the time-gating of the detected signal enabled accurate DLS measurements. This would prove to be even more powerful when the pulsed DLS scheme is combined with an infrared laser, because photodetectors with a low dark count are not readily available in the infrared region [14, 15]. Such an infrared pulsed DLS would reduce the multiple scattering from turbid dispersion, such as milk and ink [16], because the scattering intensity is inversely proportional to the 4th power of the wavelength of light [17]. The low absorption and scattering coefficients for infrared light would also increase the penetration depth in biological tissue, and thereby enable us to track the diffusion dynamics in vivo and in situ [18], which plays an important role for biological functions.

## Supplementary Information

Below is the link to the electronic supplementary material.

Supplementary file1 (DOCX 4465 KB)
