# Supplementary material for: Calibration for a count rate-dependent time correlation function and a random noise reduction in pulsed dynamic light scattering
Source: Anal Sci. 2022 Feb 15;38(3):607–11. doi: 10.1007/s44211-022-00071-0 (PMC8971174; doi:10.1007/s44211-022-00071-0)
Supplement: Supplementary file 1 — Supplementary file1 (DOCX 4465 KB) [file 44211_2022_71_MOESM1_ESM.docx]

Advancements in Instrumentation

**Supplementary material for**

**“Calibration for a count rate-dependent time correlation function and a random noise reduction in pulsed dynamic light scattering”**

Takashi HIROI,*^†^ Sadaki SAMITSU,* Hideaki KANO,** and Kunie ISHIOKA*

* *National Institute for Materials Science, 1-2-1 Sengen, Tsukuba, Ibaraki 305-0047, Japan*

** *Department of Chemistry, Faculty of Science, Kyushu University, 744 Motooka, Nishi-ku, Fukuoka 819-0395, Japan*

^†^ To whom correspondence should be addressed.

E-mail: HIROI.Takashi@nims.go.jp

**1. TEM observation experimental method and results**

**
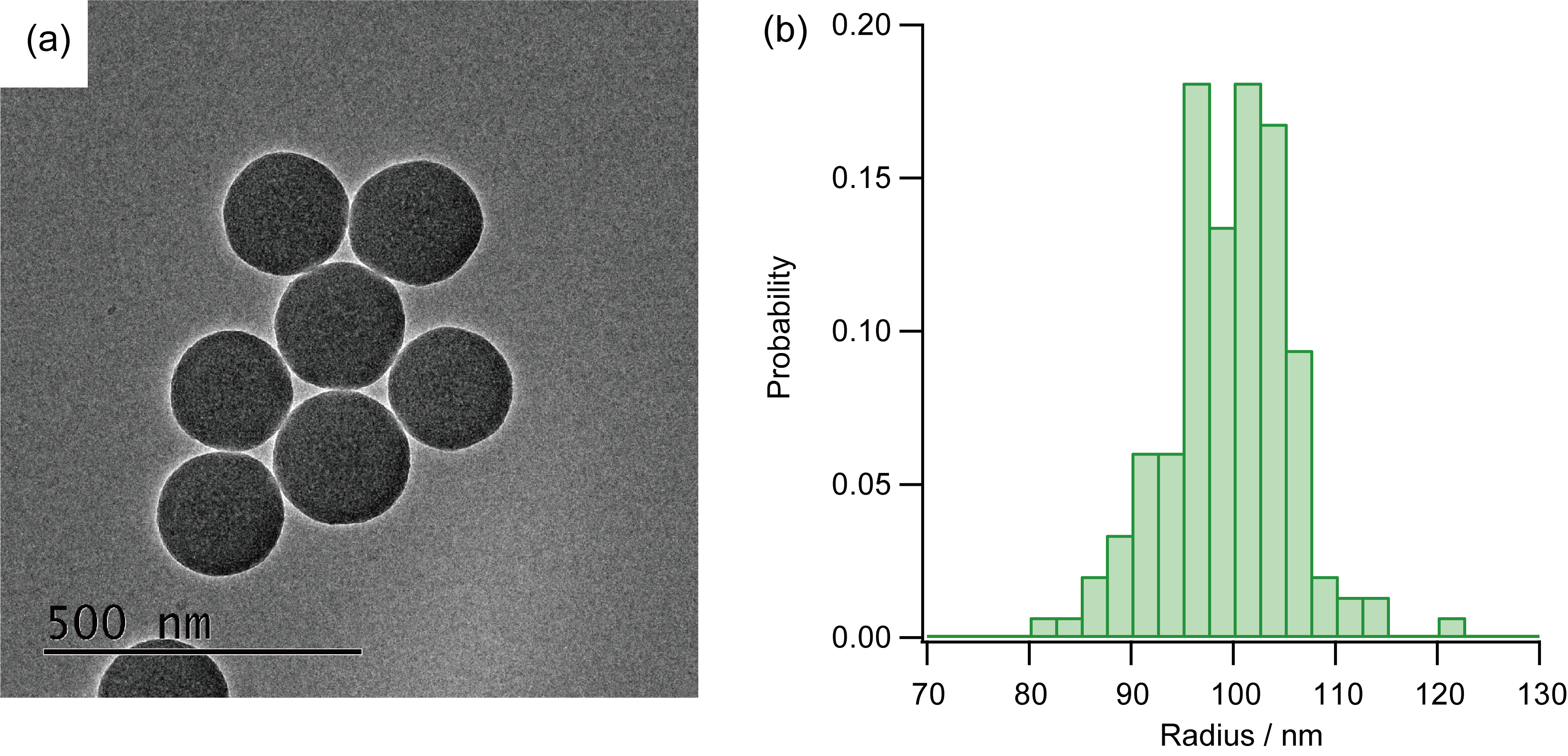
**

**Figure S1. (a) Representative TEM image of the silica nanoparticles. (b) Probability density of the observed nanoparticle radius.**

The size of the silica nanoparticles used in the present study was evaluated by transmission electron microscopy (TEM) measurements. The silica nanoparticles were drop-cast onto a TEM grid. Figure S1(a) shows a representative TEM image. The observed image was analyzed using the WinROOF software package (MITANI Corporation). Figure S1(b) shows a histogram of the probability density of the estimated radii of the silica nanoparticles (sample size *s* = 148). The average radius is estimated to be 99 ± 6 nm. To directly compare this data with DLS data, we also calculated a *Z*-average [D. Bossert *et al.*, *Anal. Chem.* **2018**, *90*, 3656.], $\left( \left\langle R^{6} \right\rangle/\langle R^{5}\rangle\right)$, which was estimated to be 101 ± 6 nm. The *Z*-average value is reported in the main text for comparison with our DLS results.

**2. Details of the numerical simulation of pulsed DLS**

To simulate the clipping effect for the dynamic light scattering (DLS) with a pulsed laser, we performed the numerical simulation based on the random walk of the particles.

First, we considered the case of DLS with a CW laser. We assumed a light electric field $E_{i}$ with a wavevector$\boldsymbol{k}_{i}$, where $\left| \boldsymbol{k}_{i} \right|=2\pi n_{r}/\lambda_{0}$ and angular frequency $\omega=c\left| \boldsymbol{k}_{i} \right|$ (where *n*_r_, *λ*_0,_ and *c* are the solvent refractive index, laser wavelength in vacuum, and speed of light, respectively), are incident onto the particles.

| $E_{i}(\boldsymbol{r},t)=E_{i}^{0}e^{i(\boldsymbol{k}_{i}\cdot\boldsymbol{r}-\omega t)}$, | (1) |
| --- | --- |

In the simulation, we used the normalized scattered electric field that does not depend on the number of particles within the irradiated volume. The normalized scattered electric field from the particles within the irradiated volume at time *t* is represented as follows:

| $E_{s}\left( t \right)=\frac{1}{\sqrt{N}}E_{i}^{0}e^{-i\omega t}\sum_{j=1}^{N} e^{i\boldsymbol{q}\cdot\boldsymbol{r}_{j}(t)}=\frac{1}{\sqrt{N}}E_{i}^{0}e^{-i\omega t}\sum_{j=1}^{N} e^{iqx_{j}(t)},$ | (2) |
| --- | --- |
| $q=\frac{4\pi n_{r}}{\lambda_{0}}\sin\frac{\theta}{2} ,$ | (3) |

where *N*, *q*, and *θ* are the number of particles within the irradiated volume, momentum transfer, and scattering angle, respectively. $\boldsymbol{r}_{j}(t)$ is the coordinate of *j*th particle and *x_j_*(*t*) is the length of $\boldsymbol{r}_{j}(t)$ projected on the direction of the momentum transfer, ${\boldsymbol{q}\equiv\boldsymbol{k}}_{i}-\boldsymbol{k}_{s}$, where $\boldsymbol{k}_{s}$ is a wavevector of scattered light. In this simulation, the fluctuation of *N,* which is essential for fluorescence correlation spectroscopy, was negligible because *N* was significantly larger than 1. In our experiment, *N* was estimated to be approximately 10^4^. From $E_{s}\left( t \right)$, the intensity of the scattered light is calculated as follows:

| $I\left( t \right)=E_{s}^{*}\left( t \right)E_{s}\left( t \right).$ | (4) |
| --- | --- |

In this definition, the time average of $I\left( t \right)$, $\left\langle I\left( t \right) \right\rangle_{T}$, converges to 1:

| $\left\langle I\left( t \right) \right\rangle_{T}=\lim_{\mathcal{T}\to+\infty} \frac{1}{\mathcal{T}}\int_{0}^{\mathcal{T}} dt\frac{1}{N}\sum_{j=1}^{N} \sum_{k\neq j}^{N} e^{iq\left( x_{j}\left( t \right)-x_{k}\left( t \right) \right)}+\frac{1}{N}\sum_{i=1}^{N} 1=1.$ | (5) |
| --- | --- |

Ideally,$I\left( t \right)$ can be measured as an intensity variation with an analog value. In the case of photon counting experiment, we measured$I\left( t \right)$ as a form of the number of photons. The average number of photons detected in *t* ~ *t* + Δ*t* was determined as follows when Δ*t* was significantly small so that the intensity variation during *t* ~ *t* + Δ*t* was negligible:

| $\left\langle n\left( t,\Delta t \right) \right\rangle_{P}=aI\left( t \right)\Delta t$ | (6) |
| --- | --- |

where *a* is the efficiency of the detection and $\left\langle\cdots\right\rangle_{P}$ stands for the averaging based on the Poisson distribution. The probability that the number of photons detected in *t* ~ *t* + Δ*t* is *n*, *P*(*n*, *t*, Δ*t*), followed the Poisson distribution:

| $P\left( n,t,\Delta t \right)=\frac{e^{{-\left\langle n\left( t,\Delta t \right) \right\rangle}_{P}}\left( \left\langle n\left( t,\Delta t \right) \right\rangle_{P} \right)^{n}}{n!}.$ | (7) |
| --- | --- |

Based on these equations, we performed a numerical simulation as follows. First, we set the detection efficiency *a* so that the preset count rate was achieved. To make the count rate equal to *X* cps, the time average of $\left\langle n\left( t,\Delta t \right) \right\rangle_{P}$ should be *X* / (1 / Δ*t*). The time average of $\left\langle n\left( t,\Delta t \right) \right\rangle_{P}$ is calculated from Eqs. (5) and (6):

| $\left\langle\left\langle n\left( t,\Delta t \right) \right\rangle_{P} \right\rangle_{T}=a\left\langle I\left( t \right) \right\rangle_{T}\Delta t=a\Delta t.$ | (8) |
| --- | --- |

From this equation, *a* is calculated to be (*X* / (1 / Δ*t*)) / Δ*t = X*. Second, we set 10^4^ particles at random positions. Based on the coordinates of the particles, the scattered intensity at *t* = 0, *I*(0), was calculated based on Eq. (4). Once we obtained the value of the scattered light intensity, we calculated $\left\langle n\left( t=0,\Delta t \right) \right\rangle_{P}$ by using Eq. (6). Third, we randomly determined the number of photons *n* detected for each time division so that the probability of the number of photons follows Poisson distribution [William H. Press *et al.*, “*Numerical recipes in C: the art of scientific computing*”, **1992**, Cambridge University Press.]. This was done by producing a random number between 0 and 1, and then converting the random number into *n* using the cumulative distribution function of Eq. (7), as schematically illustrated in Fig. S2. Finally, we moved the particles based on the theory of random walk. Whereas the diffusion in the experiments is three-dimensional (3D), Eq. (2) implies that the random walk can be treated in one dimension (1D) that is parallel to the scattering vector, ***q***, without loss of generality. This is because the diffusion whose direction is perpendicular to the momentum transfer ***q*** does not affect the value of the scattered electric field $E_{s}\left( t \right)$. We implemented both 3D and 1D calculations and confirmed that the results are the same. We therefore describe the 1D random walk in the following, where each particle moves in the positive or negative direction with equal probability. The walk step, Δ*x*, is determined as follows:

| $\Delta x=\sqrt{2D\Delta t},$ | (9) |
| --- | --- |
| $D=\frac{k_{B}T}{6\pi\eta_{s}R_{h}},$ | (10) |

where *D*, *k*_B_, *T*, *η*_s_, and *R*_h_ are the translational diffusion constant, Boltzmann constant, absolute temperature, viscosity of the solvent, and hydrodynamic radius of the particle. These values were given as parameters of the simulation with the same values as the experiment. After setting the particles to the new position, the number of photons detected in *t* = Δ*t* ~ 2Δ*t* was determined. By iterating this procedure, we obtained the time variation of the number of photons. Figure S3 shows the flow chart of this algorithm.

When we used a pulsed laser, the scattered photons were detected only during the pulse width (400 ps in the case of our system), which was significantly shorter than the dead time of the photon counting module (typically 10–100 ns). Therefore, what we recorded was not the number of photons detected in *t* ~ *t* + Δ*t* but whether a photon was detected or not. This is similar to the case of a single-clipping detection scheme [B. Chu, "*Laser Light Scattering: Basic Principles and Practice*"*,* **2007**, Dover Publications, New York, Mineola.]. To simulate the DLS with a pulsed laser, we also performed the numerical simulation with a single-clipping detection scheme by setting the number of photons detected in *t* ~ *t* + Δ*t* to be 1 if the number of photons is equal to or larger than 1. Figure S4 shows the flow chart of the algorithm with a single-clipping detection scheme.


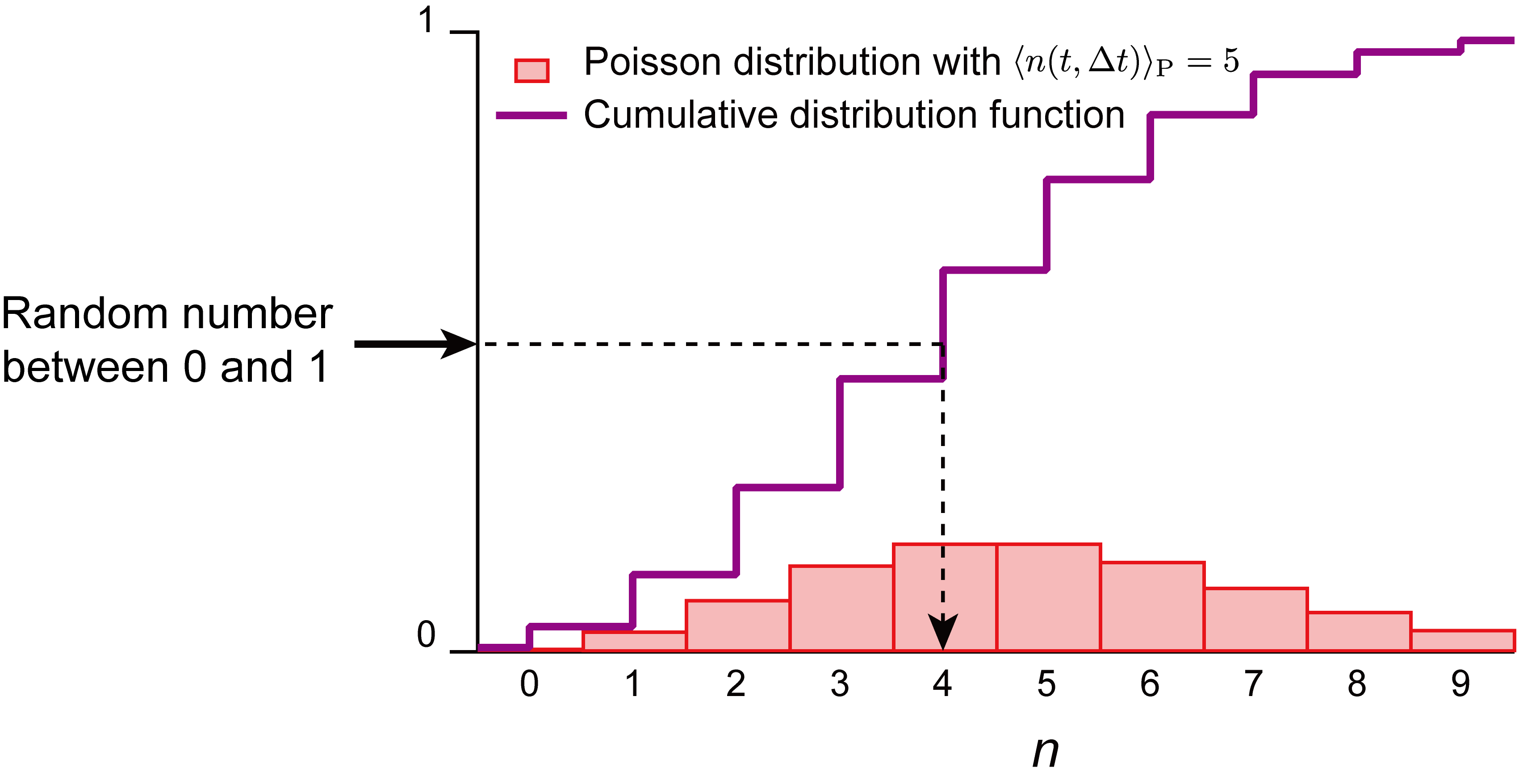


**Figure S2. Schematics of how to determine the number of detected photons that follows Poisson distribution in the case where** $\left\langle\boldsymbol{n}\left( \boldsymbol{t,}\boldsymbol{\Delta}\boldsymbol{t} \right) \right\rangle_{\mathbf{P}}\boldsymbol{=5}$**. In this case, the number of detected photons is determined to be 4 from the random number.**


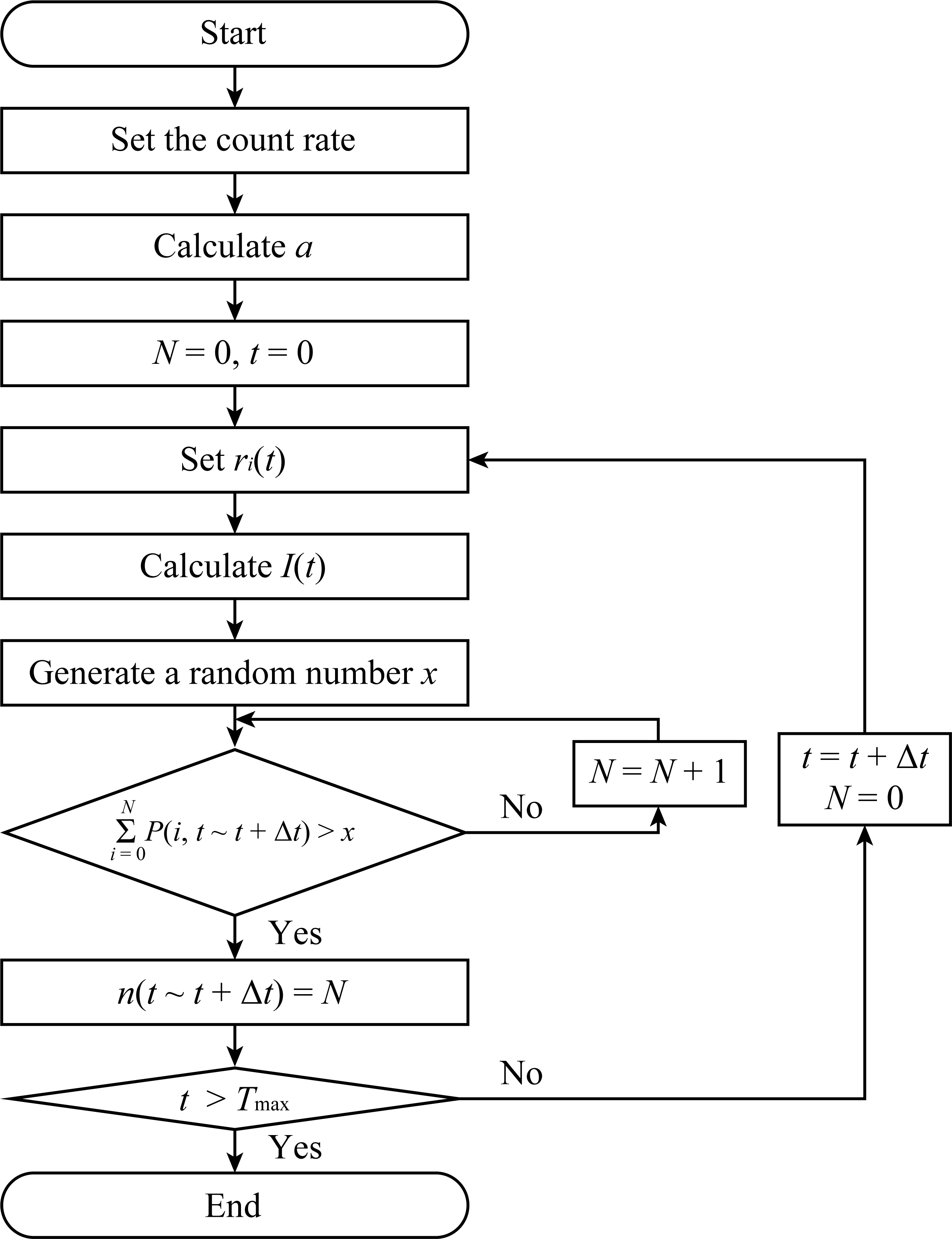


**Figure S3. Flow chart of the algorithm of the simulation of DLS without considering the clipping effect, which corresponds to the DLS with a CW laser.**


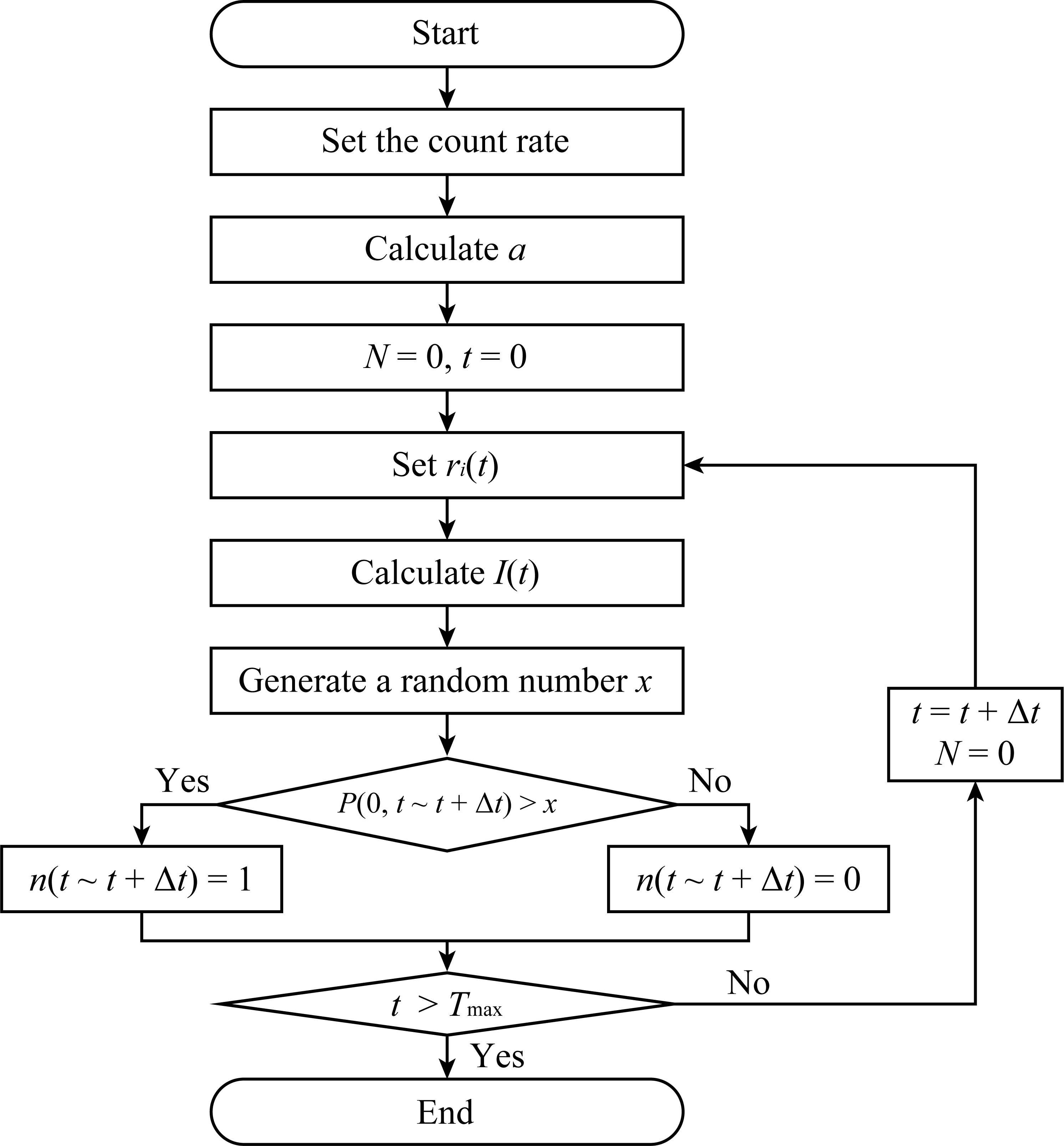


**Figure S4. Flow chart of the algorithm of the simulation of DLS with a single-clipping detection scheme, which corresponds to the DLS with a pulsed laser.**

**3. Results of the numerical simulation of pulsed DLS**

**
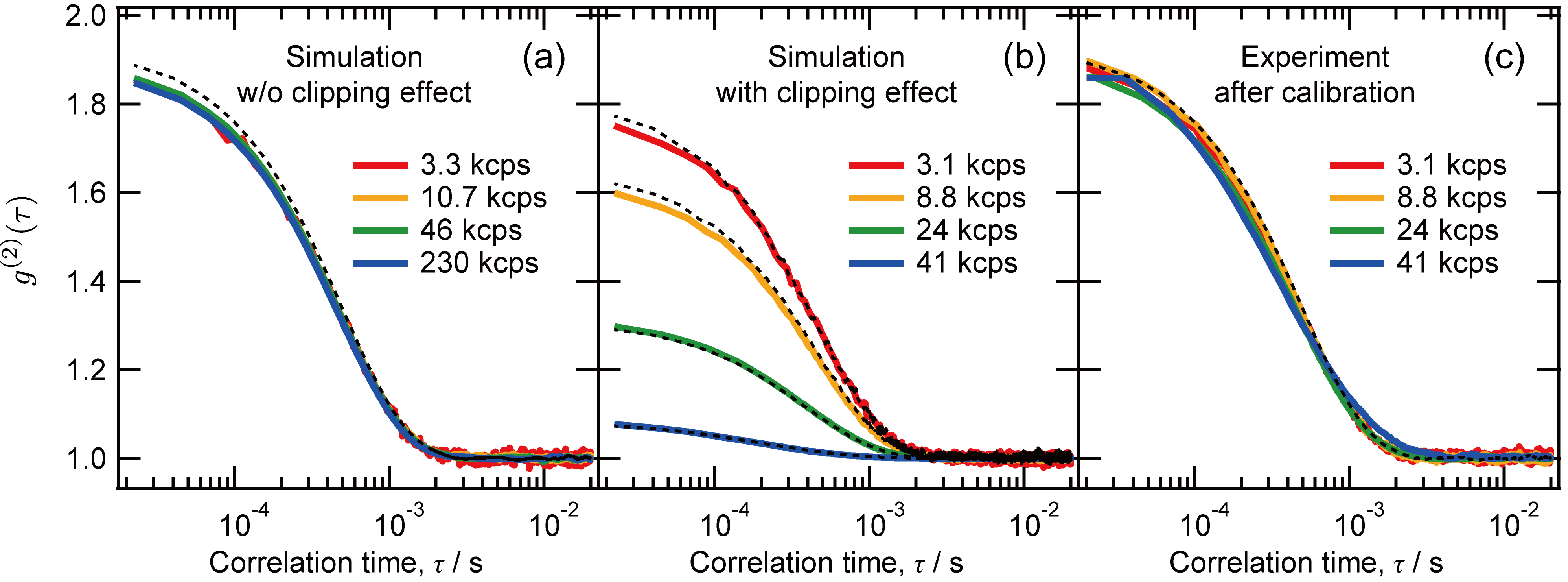
**

**Figure S5. (a), (b) Simulated time correlation functions (a) without and (b) with the clipping effect for *β*_0_ = 0.9, *R*_h0_ = 101 nm, and *θ* = 90°. The count rates of (b) are set to be the same as the experiment. Black broken lines show the corresponding experimental results obtained by (a) the CW DLS and (b) the pulsed DLS. Calculation conditions and the detection efficiencies used for the simulation of (a) are the same as that shown in (b). (c) Time correlation functions shown in Fig. 2(a) after the calibration. A black broken line shows the experimental result obtained by the CW DLS.**

**Table S1. List of correction terms** $\boldsymbol{\beta}^{\mathbf{sim}}\boldsymbol{/}\boldsymbol{\beta}_{\mathbf{0}}^{\mathbf{sim}}$**,** $\boldsymbol{D}^{\mathbf{sim}}\boldsymbol{/}\boldsymbol{D}_{\mathbf{0}}^{\mathbf{sim}}$ **and** $\boldsymbol{R}_{\boldsymbol{h}}^{\mathbf{sim}}\boldsymbol{/}\boldsymbol{R}_{\boldsymbol{h,}\mathbf{0}}^{\mathbf{sim}}$ **as a function of count rate calculated by numerical simulation. Repetition rate: *f* = 50 kHz, *N*: 1 × 10^4^, *T*_max_ = 100 s, sample size: 100.**

| **Count rate w/o clipping/cps** | **Count rate with clipping [*C*_s_]/cps** | ***C*_s_/*f*** | $\boldsymbol{\beta}^{\mathbf{sim}}\boldsymbol{/}\boldsymbol{\beta}_{\mathbf{0}}^{\mathbf{sim}}$ | $\boldsymbol{D}^{\mathbf{sim}}\boldsymbol{/}\boldsymbol{D}_{\mathbf{0}}^{\mathbf{sim}}$ | $\boldsymbol{R}_{\boldsymbol{h}}^{\mathbf{sim}}\boldsymbol{/}\boldsymbol{R}_{\boldsymbol{h,}\mathbf{0}}^{\mathbf{sim}}$ |
| --- | --- | --- | --- | --- | --- |
| 1,000 | 981(4) | 0.020 | 0.962(19) | 1.01(3) | 0.987(27) |
| 2,000 | 1,924(8) | 0.038 | 0.924(11) | 1.01(2) | 0.993(17) |
| 3,000 | 2,831(11) | 0.057 | 0.893(9) | 1.01(2) | 0.990(16) |
| 4,000 | 3,703(13) | 0.074 | 0.862(7) | 1.01(1) | 0.990(13) |
| 5,000 | 4,544(13) | 0.091 | 0.832(5) | 1.01(1) | 0.991(12) |
| 6,000 | 5,357(16) | 0.107 | 0.805(6) | 1.01(1) | 0.988(12) |
| 7,000 | 6,140(17) | 0.123 | 0.781(5) | 1.02(1) | 0.985(12) |
| 8,000 | 6,896(20) | 0.138 | 0.755(4) | 1.02(1) | 0.985(12) |
| 9,000 | 7,626(20) | 0.153 | 0.733(4) | 1.02(1) | 0.981(12) |
| 10,000 | 8,338(22) | 0.167 | 0.712(4) | 1.02(1) | 0.981(10) |
| 11,000 | 9,017(27) | 0.180 | 0.691(4) | 1.03(1) | 0.975(11) |
| 12,000 | 9,676(24) | 0.194 | 0.673(4) | 1.03(1) | 0.974(10) |
| 13,000 | 10,314(29) | 0.206 | 0.655(4) | 1.03(1) | 0.971(10) |
| 14,000 | 10,941(27) | 0.219 | 0.637(3) | 1.03(1) | 0.969(10) |
| 15,000 | 11,538(32) | 0.231 | 0.621(4) | 1.03(1) | 0.969(12) |
| 16,000 | 12,119(30) | 0.242 | 0.605(3) | 1.04(1) | 0.963(10) |
| 17,000 | 12,689(29) | 0.254 | 0.590(3) | 1.04(1) | 0.960(11) |
| 18,000 | 13,233(33) | 0.265 | 0.576(3) | 1.04(1) | 0.957(10) |
| 19,000 | 13,765(36) | 0.275 | 0.563(3) | 1.05(1) | 0.955(9) |
| 20,000 | 14,285(35) | 0.286 | 0.550(3) | 1.05(1) | 0.953(10) |
| 21,000 | 14,791(31) | 0.296 | 0.537(3) | 1.05(1) | 0.951(9) |
| 22,000 | 15,280(36) | 0.306 | 0.525(2) | 1.06(1) | 0.947(8) |
| 23,000 | 15,746(39) | 0.315 | 0.514(2) | 1.06(1) | 0.945(9) |
| 24,000 | 16,216(37) | 0.324 | 0.503(2) | 1.06(1) | 0.940(9) |
| 25,000 | 16,666(41) | 0.333 | 0.492(2) | 1.06(1) | 0.940(10) |
| 26,000 | 17,102(38) | 0.342 | 0.482(2) | 1.07(1) | 0.934(9) |
| 27,000 | 17,533(36) | 0.351 | 0.473(2) | 1.07(1) | 0.932(9) |
| 28,000 | 17,949(42) | 0.359 | 0.463(2) | 1.08(1) | 0.929(9) |
| 29,000 | 18,357(38) | 0.367 | 0.455(2) | 1.08(1) | 0.927(9) |
| 30,000 | 18,751(42) | 0.375 | 0.446(2) | 1.08(1) | 0.924(10) |
| 31,000 | 19,127(41) | 0.383 | 0.438(2) | 1.09(1) | 0.919(9) |
| 32,000 | 19,521(39) | 0.390 | 0.430(2) | 1.09(1) | 0.917(8) |
| 33,000 | 19,876(43) | 0.398 | 0.422(2) | 1.10(1) | 0.913(8) |
| 34,000 | 20,237(38) | 0.405 | 0.414(2) | 1.10(1) | 0.910(10) |
| 35,000 | 20,588(41) | 0.412 | 0.407(2) | 1.10(1) | 0.906(8) |
| 36,000 | 20,933(41) | 0.419 | 0.400(2) | 1.11(1) | 0.903(9) |
| 37,000 | 21,265(41) | 0.425 | 0.393(2) | 1.11(1) | 0.901(8) |
| 38,000 | 21,594(37) | 0.432 | 0.387(2) | 1.11(1) | 0.899(9) |
| 39,000 | 21,914(38) | 0.438 | 0.380(2) | 1.12(1) | 0.895(9) |
| 40,000 | 22,223(45) | 0.444 | 0.374(2) | 1.12(1) | 0.893(7) |
| 41,000 | 22,529(42) | 0.451 | 0.368(2) | 1.12(1) | 0.890(9) |
| 42,000 | 22,827(46) | 0.457 | 0.362(2) | 1.13(1) | 0.886(9) |
| 43,000 | 23,113(44) | 0.462 | 0.357(1) | 1.13(1) | 0.884(8) |
| 44,000 | 23,408(45) | 0.468 | 0.351(1) | 1.13(1) | 0.882(8) |
| 45,000 | 23,688(42) | 0.474 | 0.345(1) | 1.14(1) | 0.881(7) |
| 46,000 | 23,958(43) | 0.479 | 0.341(2) | 1.14(1) | 0.876(8) |
| 47,000 | 24,227(42) | 0.485 | 0.336(2) | 1.15(1) | 0.872(8) |
| 48,000 | 24,486(42) | 0.490 | 0.331(1) | 1.15(1) | 0.871(8) |
| 49,000 | 24,745(45) | 0.495 | 0.326(1) | 1.15(1) | 0.866(8) |
| 50,000 | 25,002(38) | 0.500 | 0.321(1) | 1.16(1) | 0.865(9) |
| 60,000 | 27,269(42) | 0.545 | 0.281(1) | 1.20(1) | 0.837(7) |
| 70,000 | 29,164(45) | 0.583 | 0.249(1) | 1.23(1) | 0.812(7) |
| 80,000 | 30,775(43) | 0.616 | 0.224(1) | 1.27(1) | 0.789(8) |
| 90,000 | 32,148(40) | 0.643 | 0.203(1) | 1.31(1) | 0.766(7) |
| 100,000 | 33,332(38) | 0.667 | 0.185(1) | 1.34(1) | 0.744(7) |
| 200,000 | 39,997(30) | 0.800 | 0.0969(4) | 1.69(2) | 0.593(5) |
| 300,000 | 42,853(29) | 0.857 | 0.0639(4) | 1.98(2) | 0.504(5) |
| 400,000 | 44,444(25) | 0.889 | 0.0470(3) | 2.26(2) | 0.443(4) |
| 500,000 | 45,455(20) | 0.909 | 0.0367(2) | 2.51(2) | 0.398(4) |
| 600,000 | 46,153(18) | 0.923 | 0.0299(2) | 2.74(3) | 0.365(4) |
| 700,000 | 46,664(16) | 0.933 | 0.0250(2) | 2.95(3) | 0.339(4) |
| 800,000 | 47,058(15) | 0.941 | 0.0214(2) | 3.17(3) | 0.316(3) |
| 900,000 | 47,365(14) | 0.947 | 0.0187(2) | 3.36(3) | 0.298(3) |
| 1,000,000 | 47,618(13) | 0.952 | 0.0164(1) | 3.54(4) | 0.282(3) |

**
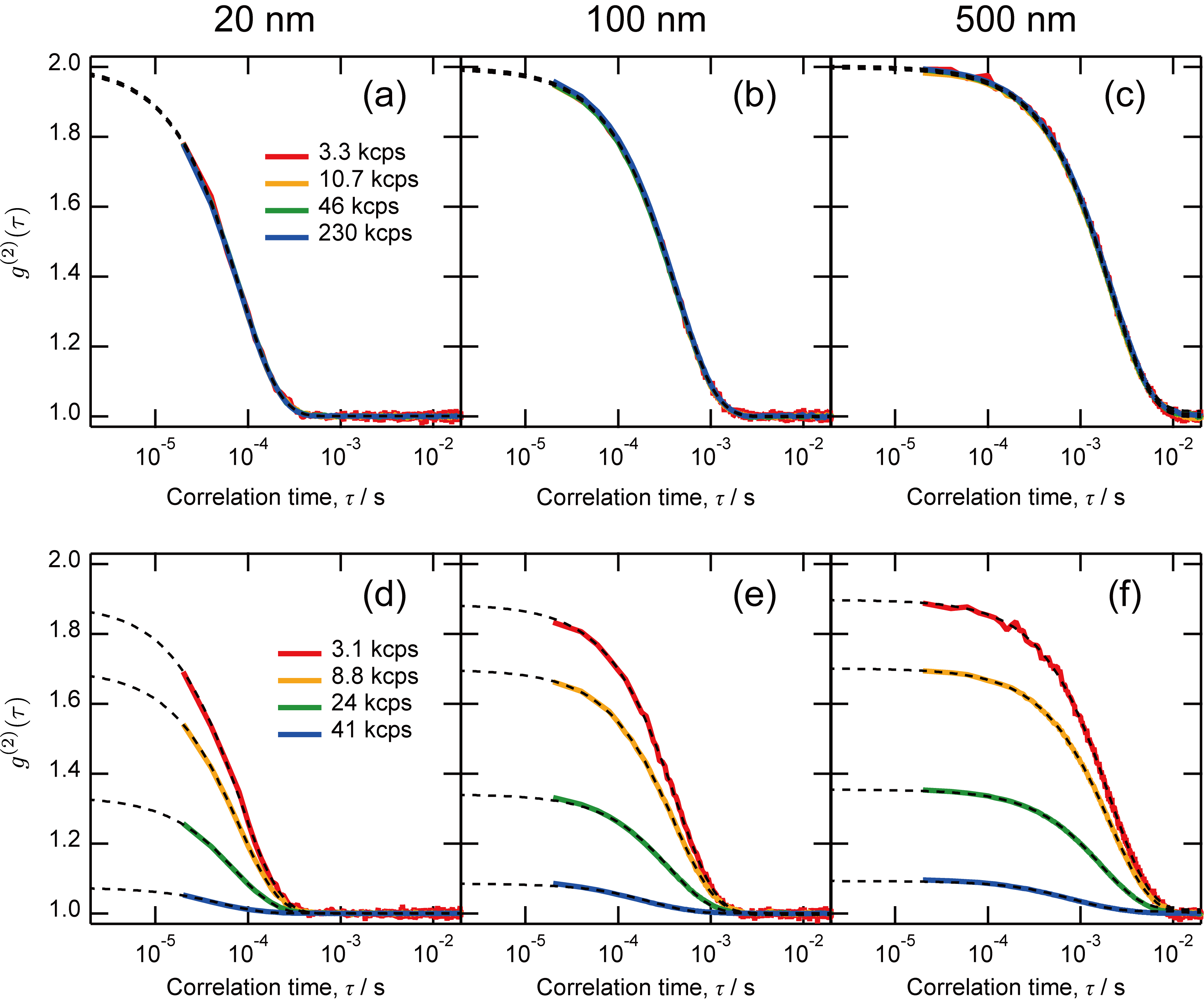
**

**Figure. S6. (a), (b), (c) Simulated time correlation functions without the clipping effect for *β*_0_ = 1 and *θ* = 90°. (a) *R*_h0_ = 20 nm, (b) *R*_h0_ = 100 nm, (c) *R*_h0_ = 500 nm. (d), (e), (f) Simulated time correlation functions with the clipping effect for *β*_0_ = 1 and *θ* = 90°. (d) *R*_h0_ = 20 nm, (e) *R*_h0_ = 100 nm, (f) *R*_h0_ = 500 nm. Black dashed lines show the fitting results based on Eq. (1) of the main text.**

**Table S2.** $\boldsymbol{\beta}^{\mathbf{sim}}\boldsymbol{/}\boldsymbol{\beta}_{\mathbf{0}}^{\mathbf{sim}}$ **and** $\boldsymbol{D}^{\mathbf{sim}}\boldsymbol{/}\boldsymbol{D}_{\mathbf{0}}^{\mathbf{sim}}$ **calculated from the simulation shown in Figure S5.**

| *R*_h0_ | $\beta^{\mathrm{sim}}/\beta_{0}^{\mathrm{sim}}$ | | | | $D^{\mathrm{sim}}/D_{0}^{\mathrm{sim}}$ | | | |
| --- | --- | --- | --- | --- | --- | --- | --- | --- |
|  | 3.1 kcps | 8.8 kcps | 24 kcps | 41kcps | 3.1 kcps | 8.8 kcps | 24 kcps | 41kcps |
| 20 nm | 0.88 | 0.70 | 0.34 | 0.07 | 1.00 | 1.02 | 1.11 | 1.53 |
| 100 nm | 0.88 | 0.70 | 0.34 | 0.08 | 0.99 | 1.02 | 1.14 | 1.75 |
| 500 nm | 0.87 | 0.69 | 0.34 | 0.09 | 1.01 | 1.03 | 1.26 | 2.34 |
